# High‐Performance Self‐Powered Perovskite Photodiodes for Blue‐Light Detection Using Anisole‐Assisted Precursor Engineering

**DOI:** 10.1002/smll.75500

**Published:** 2026-08-26

**Authors:** Kyu Young Kim, Eung Kyeong Lee, Tae Rim Ko, Young Jin Choi

**Affiliations:** ^1^ HMC Department of Nanotechnology and Advanced Materials Engineering Sejong University Seoul Republic of Korea

**Keywords:** blue light detection, perovskite, photodiode, wide bandgap perovskite

## Abstract

We report anisole‐assisted precursor engineering to realize high‐performance wide‐bandgap perovskite photodiodes for self‐powered blue‐light detection. Incorporating anisole as an internal antisolvent and applying a brief anisole bathing post‐treatment yield pinhole‐free films with enlarged grains. Under self‐powered operation (0 V), the devices achieve a low dark current density of 4.89 nA cm^−2^ and an external quantum efficiency (EQE) of 78.11% at 380 nm, corresponding to a responsivity of 0.27 A W^−1^ and a detectivity of 6.77 × 10^12^ Jones. The photodiodes maintain over 97% of their initial EQE after 1000 h of storage without encapsulation. These results highlight a practical route toward stable, self‐powered perovskite photodiodes with high sensitivity in the 350–490 nm range.

## Graphical abstract

Anisole‐assisted precursor engineering provides a simple route to high‐quality perovskite films for self‐powered blue‐light photodetection. Anisole promotes grain growth and suppresses pinholes, reducing dark current while improving external quantum efficiency and detectivity. The resulting perovskite photodiodes exhibit high sensitivity, stable operation, and long‐term durability under blue‐light illumination without external power.

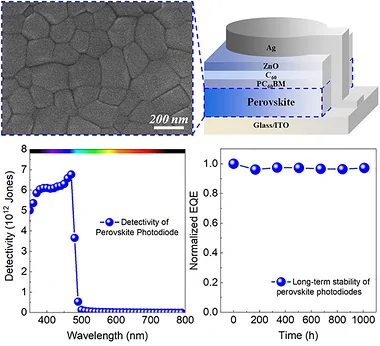

## 1 Introduction

The growing need for blue‐light detection is driven by its essential role in diverse application domains that require high spectral selectivity and sensitivity in the blue region. For example, in water quality monitoring and underwater communication [1, 2], blue wavelengths offer superior penetration depth compared with other visible wavelengths, making them particularly suitable for aquatic sensing environments. In medical diagnostics, accurate control and monitoring of blue‐light excitation is crucial for fluorescence‐based assays [3, 4] and phototherapeutic procedures [5, 6], where precise wavelength targeting directly impacts diagnostic accuracy and treatment efficacy. Furthermore, with the increasing demand for flexible and wearable optoelectronic devices, blue‐light detection has attracted substantial interest for health‐related applications, including monitoring exposure to potentially hazardous blue light [7, 8]. These diverse applications highlight the pressing demand for reliable, spectrally selective blue‐region photodetectors that combine high detectivity with long‐term operational stability.

Conventional blue‐light photodetectors are primarily based on III–V compound semiconductors such as gallium nitride (GaN), indium GaN (InGaN), and aluminum GaN (AlGaN), which exhibit excellent wide‐bandgap characteristics and strong optoelectronic performance [9, 10, 11]. However, these materials typically rely on costly and complex epitaxial growth and are generally fabricated on rigid substrates, limiting their compatibility with flexible and wearable electronics. These constraints have motivated the development of alternative photodetector materials that are low‐cost, solution‐processable, and amenable to bandgap engineering for targeting the blue spectral region.

Among the emerging candidates, organic–inorganic halide perovskites stand out due to their excellent optoelectronic properties, including high absorption coefficients, long carrier diffusion lengths, tunable bandgaps, and relatively low defect densities [12, 13, 14, 15]. Their solution‐processability enables low‐temperature, scalable fabrication on a wide range of substrates, including flexible platforms [16, 17]. Moreover, compositional tunability allows the band edge and spectral response to be tailored across a broad spectral range, from the visible to the near‐infrared, enabling high‐performance photodetectors beyond the blue region, including narrow‐bandgap Sn–Pb perovskite systems for near‐infrared (NIR) detection [18]. This spectral versatility provides a versatile route to photodetectors spanning from the blue to near‐infrared regions, with this work focusing on the blue regime [8, 19].

Despite these advantages, perovskite research has largely focused on photovoltaics and light‐emitting diodes (LED), which typically employ narrow‐to‐moderate bandgap compositions [20, 21]. In contrast, studies on wide‐bandgap perovskites specifically engineered for blue‐light detection remain comparatively limited. In particular, bandgap widening via chlorine incorporation often introduces challenges such as poor film morphology, phase instability, and increased defect densities, which collectively deteriorate photodetectors performance [19]. The absence of robust and reproducible fabrication strategies for wide‐bandgap perovskites has therefore impeded the development of efficient and stable blue‐sensitive photodetectors.

Here, we investigate a chlorine‐ and bromine‐based wide‐bandgap perovskite photodiode enabled by controlled crystallization via anisole‐assisted precursor engineering. By moving beyond conventional perovskite applications in energy harvesting and light emission, this work demonstrates the viability of wide‐bandgap perovskites for self‐powered blue‐light detection, thereby expanding the application scope of perovskite materials.

The performance of perovskite photodetectors is critically governed by thin‐film quality, including crystallinity, grain size, and defect density [22]. Uniform, pinhole‐free films with enlarged grains suppress leakage pathways and nonradiative recombination, leading to reduced dark current, enhanced photoresponse, and improved operational stability [23]. Achieving such films requires precise control over crystallization during spin coating. Antisolvent engineering is widely used to modulate nucleation and growth, thereby improving film morphology. However, conventional antisolvent dripping is highly sensitive to timing and ambient conditions, which often compromises reproducibility and scalability [24]. Furthermore, unfavorable interactions between the antisolvent and precursor solution can induce premature precipitation or heterogeneous solidification, resulting in degraded film quality [25].

In this work, we propose a simplified strategy in which anisole (a solvent with antisolvent‐like properties toward the perovskite precursor complex) is directly incorporated into the perovskite precursor solution. In addition, an anisole bathing post‐treatment is applied to reproducibly remove residual coordinating solvents, further improving film uniformity. This combined approach enables uniform modulation of crystallization during spin coating, yielding high‐quality perovskite films with pinhole‐free morphology and enlarged grains, which in turn improves both the optical and electrical characteristics of the devices. By systematically optimizing the anisole content, we achieve pronounced enhancements in dark‐current suppression, EQE, and overall photodetector performance, with the best performance obtained at 40 vol% anisole.

These results establish anisole‐assisted precursor engineering as a scalable and effective route to high‐performance wide‐bandgap perovskite photodiodes optimized for blue‐light sensing.

## 2 Results and Discussion

We improved the photoactive‐layer quality using a simple strategy involving the addition of anisole, a solvent with antisolvent‐like behavior, to the organic‐inorganic halide perovskite precursor solution. Anisole was introduced into the perovskite precursor solution, which used dimethyl sulfoxide (DMSO) as the primary solvent, at concentrations ranging from 10 to 40 vol%. The final concentration of the perovskite precursor solution was fixed at 0.9 M for all formulations. Figure 1a presents the Tauc plot analysis results for the perovskite thin films prepared with different anisole ratios. The photoactive layer maintained a constant optical bandgap of 2.58 eV across all conditions, with an absorption onset below 500 nm. These results were derived from the absorbance spectra shown in Figure S1a. The perovskite films synthesized via the antisolvent‐assisted method were integrated into a photodiode structure, as schematically illustrated in Figure 1b. The device architecture was indium tin oxide (ITO)/perovskite/[6,6]‐Phenyl‐C61‐butyric acid methyl ester (PC_60_BM)/fullerene (C_60_)/zinc oxide (ZnO)/Ag. A key feature of our design is the absence of a hole transport layer (HTL), such as poly(3,4‐ethylenedioxythiophene):poly(styrenesulfonate) (PEDOT:PSS) or poly[bis(4‐phenyl)(2,4,6‐trimethylphenyl)amine] (PTAA). This omission was intentional to reduce dark current density and to induce a voltage shift, thereby enhancing the self‐powered performance of the device. The dark current density remained stable across different applied voltages, with detailed electrical characteristics shown in Figure 2. The cross‐sectional field emission ‐ scanning electron microscopy (FE‐SEM) image (Figure 1c) confirms uniform deposition of each functional layer. We limited anisole content to 40 vol% because higher loading degraded solution/film quality (Figure S1b–d). At 50 vol% anisole, the precursor became supersaturated and visibly precipitated (Figure S1b). The film formed from the supersaturated solution showed poor coverage and non‐uniformity (Figure S1c). SEM further confirms severe pinhole formation and poor uniformity at 50 vol% anisole (Figure S1d).

**FIGURE 1:**
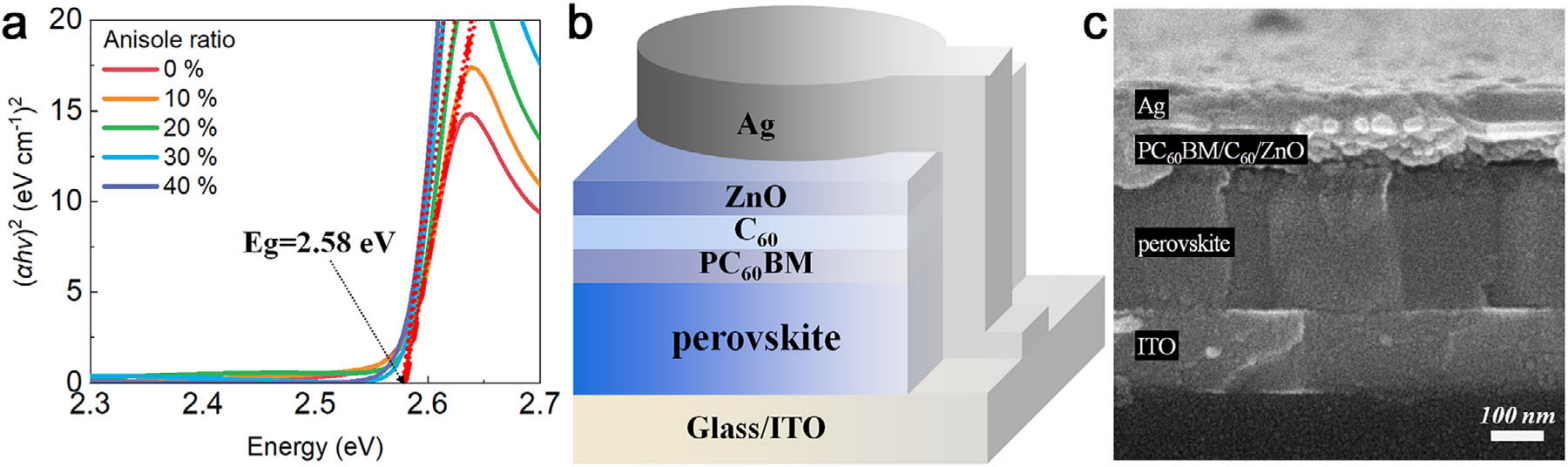
Structural and optical characterization of perovskite films and device architecture. (a) Tauc plots calculated from ultraviolet–visible–NIR (UV–vis–NIR) absorption spectra of perovskite films prepared with varying anisole contents (0–40 vol% in 10 vol% increments), indicating a consistent optical bandgap of 2.58 eV. (b) Schematic illustration of the perovskite photodiode device architecture with an ITO/perovskite/PC_60_BM/C_60_/ZnO/Ag configuration. (c) Cross‐sectional SEM image of the device, confirming the formation of well‐defined multilayer structures.

**FIGURE 2:**
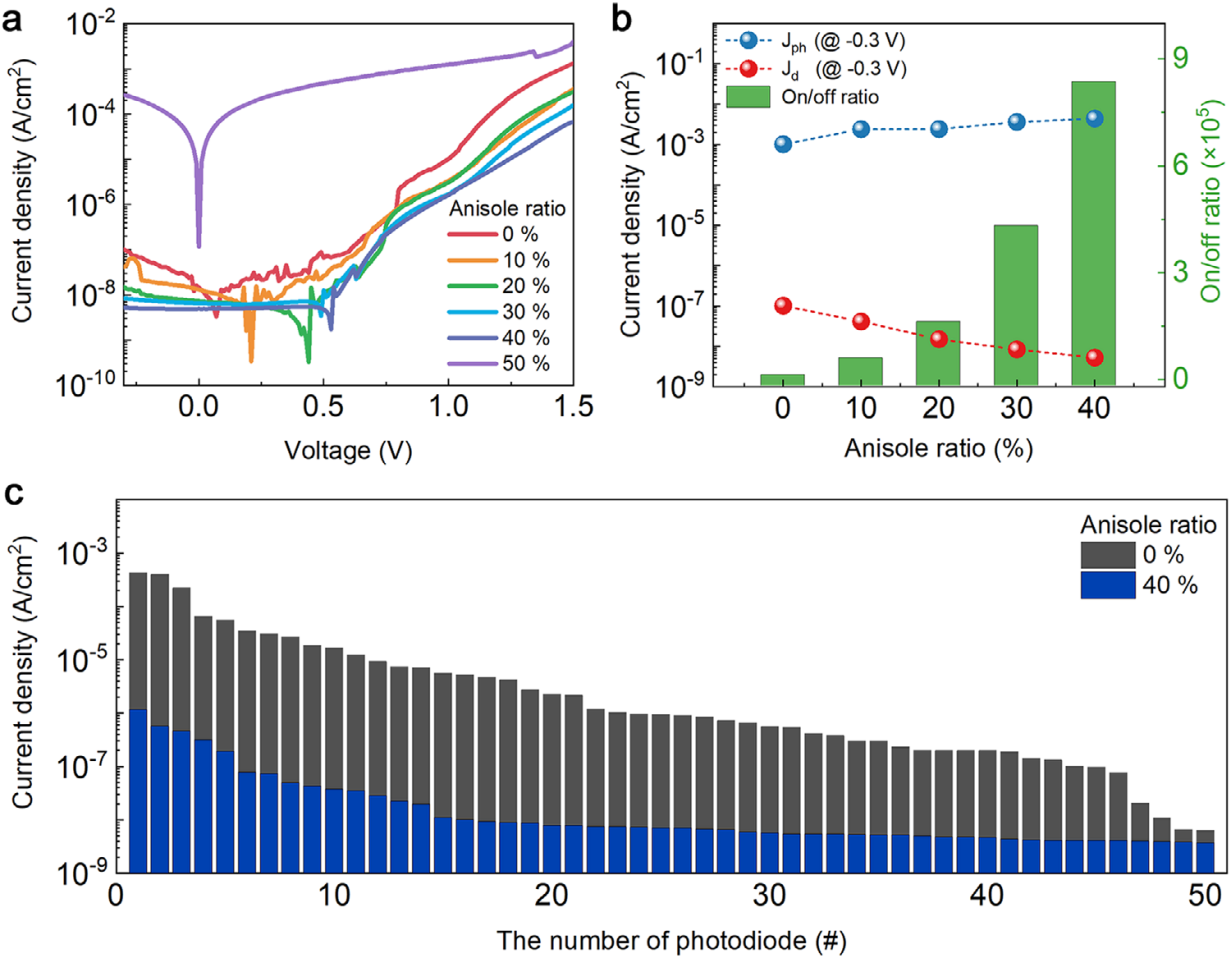
Electrical characteristics and performance of perovskite photodiodes as a function of anisole ratio. (a) Dark current density–voltage (*J–V*) characteristics, showing systematic suppression of dark current with increasing anisole content and stable operation for the device with 40 vol% anisole. (b) Summary of dark current density, photocurrent density (under 405 nm illumination), and on/off ratio at −0.3 V, demonstrating enhanced device performance and improved voltage tolerance for the 40 vol% anisole device. (c) Statistical distribution of dark current density for 50 devices fabricated without anisole (0%) and with 40 vol% anisole, demonstrating significantly improved uniformity, reproducibility, and operational stability.

Figure 2 summarizes the electrical characteristics of photodiodes fabricated via the anisole‐addition approach. Figure 2a shows the measured dark current density characteristics. The control device (0 vol% anisole) exhibits a dark current density of 16.06 nA cm^−2^ at 0 V and unstable operation, whereas increasing anisole content monotonically suppresses the dark current. Notably, the device with 40 vol% anisole shows a significantly reduced dark current density of 4.89 nA cm^−2^ at 0 V, and maintained a low value of 5.24 nA cm^−2^ even at −0.3 V, indicating improved voltage tolerance.

Figure 2b compares dark current density, photocurrent density (at 405 nm), and on/off ratio at −0.3 V for devices as a function of anisole content. Maintaining a low and stable dark current is a critical parameter in photodiode performance, especially for self‐powered devices operating at 0 V. Although many perovskite photodiodes report 0 V operation, dark current often rises sharply under small bias perturbations. To assess voltage tolerance, we measured both dark current and photocurrent at −0.3 V. Except for the unstable 50 vol% anisole device, all devices showed reduced dark current with increasing anisole content. At −0.3 V, the 40 vol% anisole device yielded the lowest dark current (5.24 nA cm^−2^), which is approximately 20 times lower than the control. The on/off ratio, calculated based on dark and photocurrent densities at 405 nm, exhibited the same trend. The 40 vol% anisole device achieved the highest on/off ratio of 8.35 × 10^5^, approximately 90 times higher than the control.

To further evaluate reproducibility and device reliability under slightly stressed conditions, Figure 2c compares dark current density of 50 devices fabricated with and without 40 vol% anisole, measured at −0.3 V. The results are arranged from highest to lowest dark current. Control devices showed large dispersion with nearly five orders of magnitude between maximum and minimum, indicating poor uniformity and stability. In contrast, approximately 70% of the devices with 40 vol% anisole exhibited dark current density in the nA cm^−2^ range. The average dark current density decreased from 2.76 × 10^−5^ to 6.63 × 10^−8^ A cm^−2^ upon 40 vol% anisole addition, demonstrating markedly improved stability. These results indicate that anisole acts as an internal antisolvent, promoting high‐quality film formation. This morphological enhancement directly contributes to improved device performance, particularly in terms of suppressed dark current and stable operation.

Figure 3 summarizes EQE measurements and related analyses of the fabricated self‐powered perovskite photodiode devices measured at 0 V. Figure 3a shows EQE spectra as a function of anisole ratio. Except for the device with 50 vol% anisole, which exhibited poor film quality and unstable photo‐response, EQE increased with increasing anisole content. Notably, the device with 40 vol% anisole exhibited EQE enhancement of approximately 1.45 times compared to the control. This device achieved a peak EQE of 78.11% at 380 nm, with an average EQE of 72.37% over the 380–470 nm spectral range.

**FIGURE 3:**
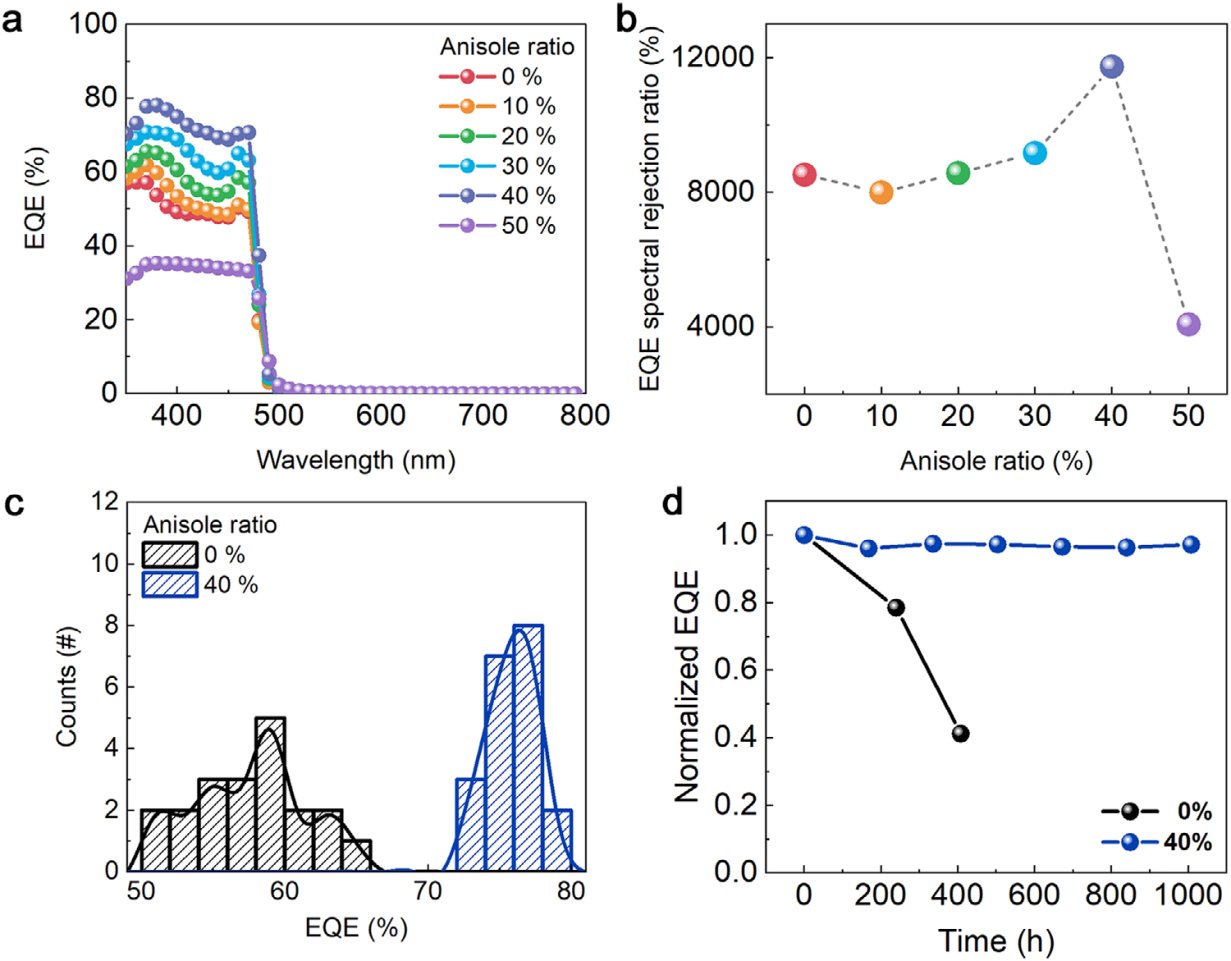
Spectral response characteristics, performance, and stability of perovskite photodiodes as a function of anisole ratio. (a) EQE spectra measured at 0 V (self‐powered operation), showing increased EQE with anisole addition and a peak EQE of 78.11% at 380 nm for the 40 vol% anisole device. (b) EQE‐based rejection ratio, calculated from EQE at 470 nm (target wavelength) and 530 nm (off‐target wavelength), indicating enhanced wavelength selectivity and an improved effective signal‐to‐noise margin for devices with increased anisole content. (c) Distribution of maximum EQE value across 20 devices measured at 0 V, demonstrating improved device‐to‐device uniformity and enhanced performance with 40 vol% anisole. (d) Normalized EQE over time for unencapsulated devices stored in an Ar‐filled glovebox, showing superior long‐term operational stability for the 40 vol% anisole device, which retains over 97% of initial EQE after 1000 h.

Figure 3b presents the EQE‐based rejection ratio as a function of anisole concentration. The rejection ratio is a key metric for wavelength‐selective photodiodes, reflecting the ability to respond to the target band while suppressing off‐band signals. Here, we define the rejection ratio as EQE(470 nm)/EQE(530 nm), where 470 nm is the target wavelength and 530 nm represents an off‐target wavelength within a 500 ± 30 nm window. The control device shows a rejection ratio of 85.25, which increased with anisole addition. The 40 vol% anisole device achieved the highest rejection ratio of 117.4, corresponding to a 1.37‐fold improvement. These results suggest efficient blue‐light harvesting with reduced parasitic response outside the target band.

Figure 3c compares the distribution of maximum EQE for 20 devices fabricated with and without 40 vol% anisole. For each device, EQE was measured from 350–780 nm, and the peak value was extracted. Control devices showed a broad distribution of peak EQE values ranging from 50% to 65%, indicating limited reproducibility and suboptimal performance. In contrast, devices with 40 vol% anisole operating as self‐powered photodiodes at 0 V consistently exhibited high peak EQE values between 73% and 79%, confirming both improved performance and strong device‐to‐device uniformity. Combined with dark‐current suppression, these results demonstrate that 40 vol% anisole yields stable optoelectronic characteristics.

Figure 3d presents normalized EQE vs. time to evaluate long‐term operational stability. All devices were stored in an Ar‐filled glovebox without additional encapsulation. Full time‐dependent EQE data are provided in Figure S2. Control devices degraded substantially, with EQE falling below 50% of the initial value after 400 h. In contrast, devices with 40 vol% anisole retained over 97% of their initial EQE even after 1000 h, indicating excellent operational stability.

We analyzed the performance of perovskite photodiodes as a function of the anisole addition ratio and confirmed that devices fabricated with 40 vol% anisole exhibited improved electrical and optical characteristics. To validate the enhanced quality of perovskite thin films with 40 vol% anisole, we performed morphological and structural analyses.

Figure 4a,b present the SEM images of perovskite films fabricated without anisole and with 40 vol% anisole, respectively. The film prepared without anisole exhibited numerous pinholes and small grains, indicating poor uniformity. By contrast, the film synthesized with 40 vol% anisole addition was pinhole‐free and comprised markedly larger grains. These trends, reduction in pinholes and grain growth with increasing anisole ratio, are consistently observed in the SEM images for various anisole concentrations (see Figure S3a–c). Figure 4c shows grain size distributions extracted from SEM analysis. In the control film, most grains were below 200 nm in size, with a peak at approximately 100 nm. In contrast, the film with 40 vol% anisole exhibited a broader distribution, with the largest grains exceeding 300 nm. The presence of pinholes in perovskite layers is known to contribute to leakage currents, degrading the performance of photodiodes. Additionally, larger grains reduce grain‐boundary density, which commonly serves as non‐radiative recombination sites and leakage paths. Accordingly, anisole‐treated films with improved morphology and larger grains enable lower dark current and more efficient charge collection, yielding enhanced photoresponse.

**FIGURE 4:**
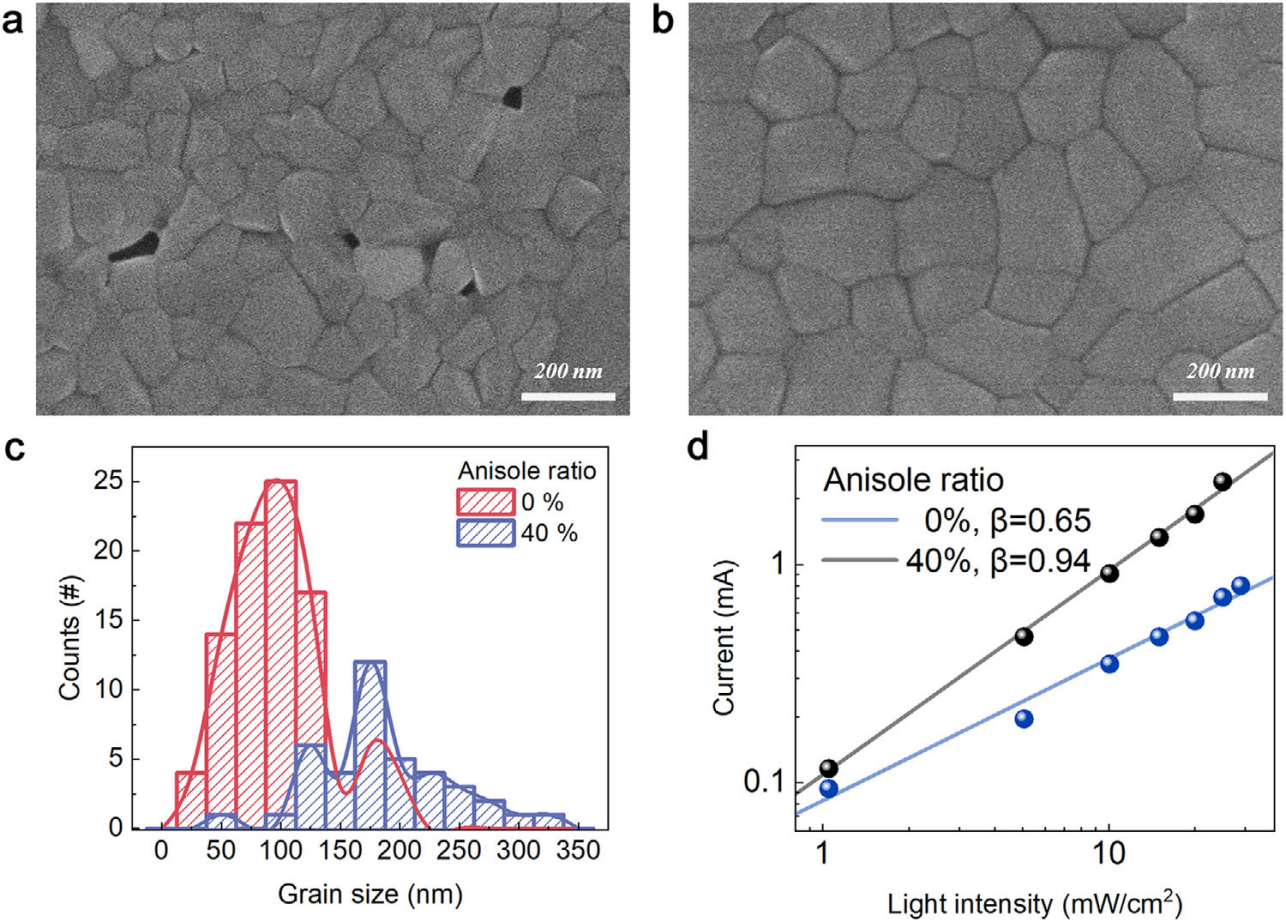
Morphological and structural characterization of perovskite films as a function of anisole ratio. (a,b) Top‐view SEM images of perovskite films fabricated without anisole (0 vol%) and with 40 vol% anisole. (c) Grain size distributions derived from SEM analyses, showing larger grains exceeding 300 nm for the film with 40 vol% anisole, compared to predominantly sub‐200 nm grains for the film without anisole. (d) Light‐intensity‐dependent short‐circuit current (*I_SC_*) measurements plotted on a log–log scale and fitted with the power law *I_SC_* ∝ *P^β^*, demonstrating efficient carrier extraction (*β* = 0.94) and low defect‐related recombination in the 40 vol% anisole device, in contrast to sublinear behavior (*β* = 0.65) for the device without anisole.

To further validate the reduction in defect density associated with increased grain size, we performed light‐intensity‐dependent short‐circuit current (*I*_SC_) measurements. Figure 4d displays the power‐law fitting of *I*_SC_ vs. incident light power *I*_SC_ (*I_SC_* ∝ *P^β^*), based on the *I*–*V* data in Figure S3d,e. The control device exhibited a *β* value of 0.65, far below unity. This sublinear scaling likely arises from space‐charge effects or interfacial carrier imbalance, which are commonly associated with a higher defect density [19, 26]. In contrast, the device with 40 vol% anisole yielded *β* value of 0.94, close to unity, indicating efficient carrier extraction with reduced defect‐mediated recombination. These findings support that 40 vol% anisole produces lower‐defect and higher‐quality films, directly contributing to improved photodiode performance.

The formation of high‐quality perovskite films with large grain sizes and pinhole‐free morphology is primarily governed by precursor solvent selection, processing conditions during spin coating, and solvent evaporation behavior during early‐stage crystallization. Precursor solvent selection plays a central role in perovskite film formation, as the solubility and coordination behavior of methylammonium bromide (MABr), methylammonium chloride (MACl), lead(II) bromide (PbBr_2_), and lead(II) chloride (PbCl_2_) strongly depend on the solvent, thereby influencing nucleation density and early‐stage crystal growth [27]. DMSO was employed as the primary solvent due to its strong coordination toward metal halides and moderate volatility, enabling uniform precursor deposition without premature crystallization. Although excessive anisole (50%) caused precipitation, anisole contents below 40% preserved solution stability while enabling controlled early‐stage crystallization (Figure S1b).

During spin coating, solvent evaporation dynamics critically determine nucleation kinetics and the transient precursor morphology. To mitigate radial solvent evaporation gradients and the resulting non‐uniform crystallization, we physically fixed the substrate during spin coating, which yielded more radially symmetric island‐like precursor morphologies [25]. Under these controlled conditions, the effect of anisole on precursor morphology is evident in Figure 5a,b. Spin coating from pure DMSO produces small and poorly connected islands, whereas anisole incorporation leads to larger and more uniformly distributed islands. Increasing anisole content from 10 to 30 vol% progressively enlarges island size (Figure S4a–c), indicating modified nucleation behavior and regulated early‐stage crystallization.

**FIGURE 5:**
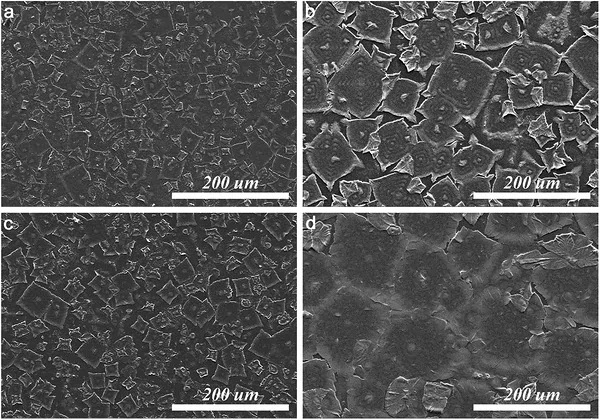
SEM analysis of transient precursor morphology and annealed film microstructure with and without anisole. (a,b) SEM images of precursor films immediately after spin coating from (a) DMSO‐only and (b) DMSO–anisole (40 vol%) precursor solutions, showing larger and more uniform islands upon anisole addition. (c,d) SEM images after thermal annealing, showing the formation of larger, well‐connected crystal domains morphology for the 40 vol% anisole film compared with the DMSO‐only film. These results suggest that anisole‐assisted solvent engineering modulates early‐stage crystallization, leading to compact, pinhole‐free films and improved photodiode performance. For the samples in Figure 5, the bathing step was omitted.

These early‐stage morphological features are reflected into the final annealed film characteristics. As shown in Figure 5c,d, anisole‐added films form larger and better‐connected crystal domains than the DMSO‐only film. Under the present processing conditions, DMSO‐only films exhibit rapid solvent loss that promotes abrupt nucleation and heterogeneous crystal growth, whereas anisole modifies solvent evaporation dynamics and slows the evolution of supersaturation and promotes more homogeneous crystallization leading to a more gradual crystallization process consistent with antisolvent‐assisted crystallization mechanisms [24]. Accordingly, the anisole‐containing system exhibits a more uniform crystallization process (Figure 5d) directly translates into the compact, pinhole‐free morphology and enlarged grain structure observed in the final annealed films (Figure 4b), supporting the link between early‐stage crystallization control and final film quality.

Taken together, Figures 4 and 5 demonstrate that anisole‐assisted solvent engineering enables controlled early‐stage crystallization and modulates grain growth by regulating solvent evaporation dynamics during spin coating, providing a microstructural basis for the reduced dark current density and improved optoelectronic performance of the resulting perovskite photodiodes.

Figure 6a,b summarize responsivity (*R*) and detectivity (D^*^), two key photodiode performance metrics. Responsivity was calculated from the measured EQE under self‐powered operation (0 V) using Equation 1:

(1) $$R=EQE\times \left(q/h\nu \right)$$

where *q* is the elementary charge, *h* is Planck's constant, and ν is the frequency of incident light. Since responsivity is directly calculated from EQE, it exhibits the same increasing trend with anisole content. The maximum responsivity observed for the photodiode with 40 vol% anisole was 0.27 A W^−1^.

**FIGURE 6:**
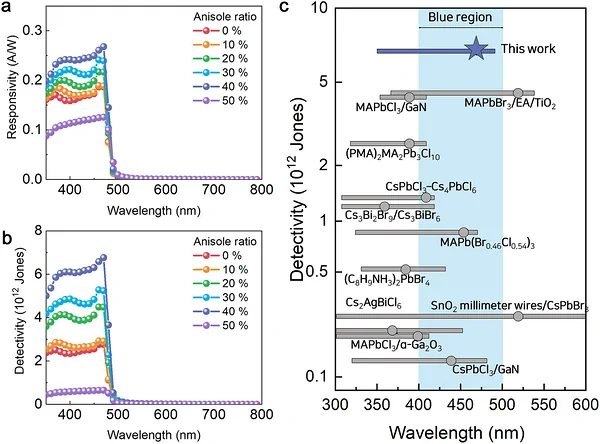
Responsivity and detectivity of perovskite photodiodes with varying anisole ratios. (a) Calculated responsivity (*R*) from EQE measured at 0 V (self‐powered operation), showing a maximum of 0.27 A W^−1^ for the 40 vol% anisole device and a systematic increase with higher anisole content. (b) Detectivity (D^*^) calculated using responsivity and dark current density measured at 0 V, reaching 6.77 × 10^1^ ^2^ Jones for the 40 vol% anisole device, indicating high sensitivity. (c) Comparison of peak detectivity and detection wavelength range of the 40 vol% anisole perovskite photodiode with previously reported blue‐light photodetectors, based on data summarized in Table S1, demonstrating enhanced performance and color selectivity.

Detectivity was estimated using Equation (2) based on the responsivity and dark current measured at 0 V under self‐powered operation, as shown in Figure 6c:

(2) $$D^{*}=\frac{R}{\sqrt{2qJ_{d}}}$$

where *J_d_* is the dark current density. This expression corresponds to a shot‐noise‐based approximation, assuming that shot noise dominates the total noise current. Therefore, the calculated D^*^ a shot‐noise‐limited estimate of the device detectivity based on the measured dark current density. Because D^*^ accounts for both responsivity and dark current, it provides a useful comparative metric for evaluating device performance. The device fabricated with 40 vol% anisole exhibited superior and stable performance in both parameters, achieving a maximum estimated detectivity of 6.77 × 10^12^ Jones.

To evaluate the performance of our proposed perovskite photodiode for blue light detection, we compared it with previously reported studies. Figure 6c and Table S1 show the peak detectivity and detection wavelength range of our photodiode (with 40 vol% anisole) in comparison to various blue photodetectors, including organic, inorganic, and composite materials. To the best of our knowledge, our device shows a higher detectivity than many previously reported blue photodetectors. With its excellent color selectivity in the blue‐light region (350 to 490 nm), our device is well suitable for blue‐light detection applications.

## 3 Conclusion

In summary, incorporating anisole into the perovskite precursor as an internal antisolvent, together with a brief bathing post‐treatment, provides a simple route to improve film quality and photodiode performance. At the optimized 40 vol% anisole condition, the films become pinhole‐free with enlarged grains, reducing leakage pathways and enabling stable self‐powered operation. Consequently, the photodiodes exhibit a low dark current density of 4.89 nA cm^−2^ at 0 V and a high EQE of 78.11% at 380 nm at 0 V, delivering a responsivity of 0.27 A W^−1^ and a detectivity of 6.77 × 10^12^ Jones. The devices retain over 97% of the initial EQE after 1000 h of storage without encapsulation, indicating strong operational stability. Beyond the present device architecture, this anisole‐assisted precursor engineering strategy should be broadly applicable to wide‐bandgap perovskite photodiodes, offering a practical route toward self‐powered UV/blue optoelectronic applications

## 4 Experimental Section

### 4.1 Materials

All chemicals were used as received without further purification. MABr (>99.99%), MACl (>99.99%), and formamidinium chloride (FACl, >99.99%) were purchased from GreatCell Solar Materials. PbBr_2_ (>98.0%) and PbCl_2_ (>99.5%) were purchased from Tokyo Chemical Industry. DMSO (>99.9%, anhydrous), anisole, chlorobenzene, alconox detergent, acetone, and isopropyl alcohol (IPA) were purchased from Sigma–Aldrich. C_60_ (99.5%) and PC_60_BM (>99.0%) were purchased from 1‐Material. ZnO nanoparticle (NP) was purchased from Nano clean tech.

### 4.2 Fabrication of Perovskite Thin Films

The perovskite precursor solution was prepared by dissolving MABr, MACl, FACl, PbBr_2_, and PbCl_2_ in DMSO to obtain the nominal composition of FA_0.4_MA_0.6_Pb(Br_0.55_Cl_0.45_)_3_. The solution was stirred at room temperature for 1 h and filtered through a 0.2 µm poly(tetrafluoroethylene) (PTFE) membrane before use. Anisole was incorporated at 10%, 20%, 30%, 40%, and 50% by volume.

Perovskite films were coated in an argon‐filled glovebox using a spin‐coater (OSSILA L2001B2‐U2‐EU) that physically secures the substrate during spinning. The precursor solution was spin‐coated at 4000 rpm for 20 s, followed by immersion in an anisole bath for 60 s to extract residual solvent from the film. After the bathing step, the substrates were spun at 4000 rpm for 4 s to remove any remaining anisole from the film surface. Sequential thermal annealing was then performed at 60^°^C for 3 min and 100°C for 10 min. For the films shown in Figure 5 and Figure S4, the same procedure was followed except that the bathing process was omitted.

### 4.3 Fabrication of Perovskite Photodiodes

The patterned ITO substrate was sequentially cleaned in an ultrasonic bath for 10 min each with alconox detergent solution (dissolved in deionized water), deionized water (twice), acetone, and IPA. The substrates were then dried under a nitrogen (N_2_) gas stream and treated with UV–O_3_ for 30 min. The perovskite layers were spin‐coated as described above.

PC_60_BM (5 mg/mL in chlorobenzene) was spin‐coated onto the perovskite layer at 6000 rpm for 40 s, followed by thermal annealing at 100°C for 1 min. A 30 nm C_60_ layer was deposited by thermal evaporation under vacuum. The ZnO NP layer was deposited by drop‐casting the solution onto the C_60_ layer, followed by spinning at 6,000 rpm to ensure uniform coverage. Finally, a 100 nm silver electrode was deposited via thermal evaporation under vacuum.

### 4.4 Characterization and Device Measurements

UV–vis–NIR absorption spectra were measured using a Cary 5000 spectrometer (Agilent Technologies). Top‐view and cross‐sectional FE‐SEM images were obtained using a HITACHI SU‐8010. Photodetector *J–V* characteristics were measured using Keithley 4200 and 2450 source meters under dark and illuminated conditions (405 nm blue laser diode, variable light intensities). The device active area was 0.2 cm^2^. EQE, spectral responsivity, and spectral detectivity were measured using a tunable light source for QE (IQE‐200, Newport).

## Author Contributions

**Kyu Young Kim**: Conceptualization, Data curation, Formal analysis, Visualization, Writing – original draft, Writing – review and editing. **Eung Kyeong Lee**: Methodology, Investigation, Data curation, Validation, Formal analysis, Visualization, Writing – original draft, **Tae Rim Ko**: Investigation, Validation. **Young Jin Choi**: Conceptualization, Supervision, Project administration, Funding acquisition, Resources, Writing – review and editing.

## Funding

This research was supported by Nano & Material Technology Development Program through National Research Foundation of Korea(NRF) funded by Ministry of Science and ICT (No. 2022M3H4A1A04096380 (RS‐2022‐NR068226)), and Basic Science Research Program through NRF funded by Ministry of Education (No. NRF‐2020R1A6A1A03043435).

## Conflicts of Interest

The authors declare no conflicts of interest.

## Supporting information




**Supporting File**: smll75500‐sup‐0001‐SuppMat.docx.

## Data Availability

The data that supports the findings of this study are available in the supplementary material of this article.
