# Supplementary material for: High‐Performance Self‐Powered Perovskite Photodiodes for Blue‐Light Detection Using Anisole‐Assisted Precursor Engineering
Source: Small. 2026 Aug 26;22(62):e75500. doi: 10.1002/smll.75500 (PMC13742902; doi:10.1002/smll.75500)
Supplement: Supplementary file 1 [file SMLL-22-e75500-s001.docx]

Supporting Information

High-Performance Self-Powered Perovskite Photodiodes for Blue-Light Detection Using Anisole-Assisted Precursor Engineering

Kyu Young Kim, Eung Kyeong Lee, Tae Rim Ko and Young Jin Choi^*^

HMC, Department of Nanotechnology and Advanced Materials Engineering, Sejong University, Seoul, 05006, Republic of Korea

E-mail: [jini38@sejong.ac.kr](mailto:jini38@sejong.ac.kr)

[+] K.Y. Kim and E.K. Lee contributed equally to this work.

**Figure S1**

Optical and visual characterization of perovskite films with different anisole content. **a**. UV–Vis-NIR absorption spectra of perovskite thin films with varying anisole ratios. **b**. Photographs of precursor solutions. The 50 vol% anisole solution appears turbid and shows partial precipitation, whereas lower concentrations remain clear. **c**. Photograph of the film fabricated from 50 vol% anisole solution. The surface is non-uniform and partially opaque. **d**. Top-view SEM image of the 50 vol% anisole film. The morphology is degraded with visible surface defects due to precipitation.

**Figure S2**

Long-term EQE stability of perovskite photodiodes measured at 0 V. **a**. Time-dependent EQE of a device without anisole (0%) measured up to 408 hours, showing significant degradation over time. **b**. Time-dependent EQE of a device with 40 vol% anisole measured up to 1008 hours, demonstrating excellent stability with minimal loss in EQE. **c**. Maximum EQE of devices without and with 40 vol% anisole plotted over time, demonstrating operational stability for the 40 vol% anisole device.

**Figure S3**

Film morphology and photocurrent response of devices. **a–c**. Top-view SEM images of perovskite films with 10 vol%, 20 vol%, and 30 vol% anisole. **d**, **e**. Photocurrent density (*J_p_* at 405 nm) of devices fabricated without anisole and with 40 vol% anisole.

**Figure S4**

Morphology of perovskite films before and after thermal annealing. **a–c**. SEM images of perovskite precursor films immediately after spin coating with 10 vol%, 20 vol%, and 30 vol% anisole. The size of the islands increases with higher anisole content. **d–f**. SEM images of the corresponding films after thermal annealing. The domain size remains consistent with the trend observed in the precursor films, increasing as the anisole content rises.

| Materials | Range  (nm) | Peak *D**  (nm) | Detectivity  (10^12^ Jones) | Noise model | Bias condition | Structure  type | Device area | Reference |
| --- | --- | --- | --- | --- | --- | --- | --- | --- |
| FA_0.4_MA_0.6_Pb(Br_0.55_Cl_0.45_)_3_ | 350-490 | 470 | 6.77 | Dark-current limited | 0 V | Vertical | 0.2 cm^2^ | This  work |
| MAPbBr_3_/EA/TiO_2_ | 370-540 | 520 | 4.46 | Dark-current limited | 0 V | Lateral | 0.0006 cm^2^ | S1 |
| MAPbCl_3_/GaN | 355-410 | 390 | 4.30 | Shot-noise limited | 5 V | Lateral | 0.00036 cm^2^ | S2 |
| (PMA)_2_MA_2_Pb_3_Cl_10_ | 320-410 | 390 | 2.59 | Dark-current limited | 0.5 V | Vertical | 0.0314 cm^2^ | S3 |
| CsPbCl_3_–Cs_4_PbCl_6_ | 310-420 | 410 | 1.35 | Dark-current limited | 0 V | Vertical | 0.09 cm^2^ | S4 |
| Cs_3_Bi_2_Br_9_/Cs_3_BiBr_6_ | 310-420 | 360 | 1.20 | Dark-current limited | 0 V | Vertical | 0.000025 cm^2^ | S5 |
| MAPb(Br_0.46_Cl_0.54_)_3_ | 325-470 | 455 | 0.87 | Dark-current limited | 0 V | Vertical | 0.348 and 0.08 cm^2^ | S6 |
| (C_8_H_9_NH_3_)_2_PbBr_4_ | 330-430 | 385 | 0.52 | Dark-current limited | 10 V | Vertical | 0.00704 cm^2^ | S7 |
| SnO_2_millimeterwires/ CsPbBr_3_ | 300-600 | 520 | 0.26 | Dark-current limited | 3 V | Lateral | 0.000534 cm^2^ | S8 |
| Cs_2_AgBiCl_6_(CABC) | 300-450 | 370 | 0.21 | Shot-noise limited | 3 V | Vertical | 0.017 cm^2^ | S9 |
| MAPbCl_3_/α-Ga_2_O_3_ | 300-410 | 400 | 0.19 | Dark-current limited | 0 V | Vertical | 0.04 cm^2^ | S10 |
| CsPbCl_3_/GaN | 320-480 | 440 | 0.13 | Dark-current limited | -11 V | Vertical | 0.00005 cm^2^ | S11 |

**Table S1**

Summary of reported blue-light photodetectors used for comparison. The table includes materials, detection wavelength range, peak wavelength for maximum detectivity, peak detectivity (*D**), noise model, bias condition, device structure type, and device area for our 40 vol% anisole perovskite photodiode and previously reported devices. This information is provided to ensure a consistent basis for comparison across different studies.

**References**

S1.	A. A. Khan, Z. Yu, U. Khan, L. Dong, Solution Processed Trilayer Structure for High-Performance Perovskite Photodetector. *Nanoscale Research Letters* **2018**, *13*, 399.

S2.	S. Jiang, W. Wei, S. Li, Y. Tian, Y. Yun, M. Chen, R. Zhang, Perovskite/GaN-Based Light-Modulated Bipolar Junction Transistor for High Comprehensive Performance Visible-Blind Ultraviolet Photodetection. *ACS Photonics* **2024**, *11*, 3026–3036.

S3.	J. Han, C. Liu, Y. Zhang, Y. Guan, X. Zhang, W. Yu, L. Xiao, Quasi‐2D Ruddlesden–Popper Perovskites with Low Trap‐States for High Performance Flexible Self‐Powered Ultraviolet Photodetectors. *Advanced Optical Materials* **2022**, *10*, 2201431.

S4.	W. Zhu, M. Deng, D. Chen, Z. Zhang, W. Chai, D. Chen, Y. Hao, Dual-Phase CsPbCl_3_-Cs_4_PbCl_6_ Perovskite Films for Self-Powered, Visible-Blind UV Photodetectors with Fast Response. *ACS Applied Materials & Interfaces* **2020**, *12*, 32961–32969.

S5.	F. Cao, T. Yan, Z. Li, L. Wu, X. Fang, Dual-Band Perovskite Bulk Heterojunction Self-Powered Photodetector for Encrypted Communication and Imaging. *Advanced Optical Materials* **2022**, *10*, 2200786.

S6.	Y. Yun, H. Cho, J. Jung, S. W. Yang, D. Vidyasagar, R. K. Gunasekaran, S. Lee, High-performance self-powered color filter-free blue photodetector based on wide-bandgap halide perovskites. *Journal of Materials Science & Technology* **2023**, *152*, 100–108.

S7.	Y. Li, D. Kuang, J. Guo, B. Liu, Y. Zhang, S. Xu, Z. Yu, Wavelength selective and cesium halides additive photodetectors based on two-dimensional perovskite: (C_8_H_9_NH_3_)_2_PbBr_4_. *Journal of Alloys and Compounds* **2022**, *904*, 163990.

S8.	Y. Zhang, W. Xu, X. Xu, J. Cai, W. Yang, X. Fang, Self-Powered Dual-Color UV–Green Photodetectors Based on SnO_2_ Millimeter Wire and Microwires/CsPbBr_3_ Particle. *The Journal of Physical Chemistry Letters* **2019**, *10*, 836–841.

S9.	S. Jiang, R. Huang, W. Li, X. Huang, H. Sheng, F. Wu, W. Mai, Low-Temperature Vapor-Phase Anion-Exchange Strategy for Wide-Bandgap Double-Perovskite Cs_2_AgBiCl_6_ Films toward Weak Ultraviolet Light Imaging. *ACS Applied Materials & Interfaces* **2022**, *14*, 26279–26286.

S10. S. Liu, S. Jiao, J. Zhang, H. Lu, D. Wang, S. Gao, L. Zhao, High-Detectivity and sensitive UVA photodetector of polycrystalline CH_3_NH_3_PbCl_3_ improved by α-Ga_2_O_3_ nanorod array. *Applied Surface Science* **2022**, *571*, 151291.

S11. H. Liu, B. Ye, Y. Gu, Y. Liu, X. Yang, F. Xie, G. Yang, UV–visible dual-band photodetector based on an all-inorganic Mn-doped CsPbCl_3_/GaN type-II heterojunction. *Applied Physics Letters* **2023**, *123*, 232105.
